# Body-Mass Scaling of Metabolic Rate: What are the Relative Roles of Cellular *versus* Systemic Effects?

**DOI:** 10.3390/biology4010187

**Published:** 2015-03-04

**Authors:** Douglas S. Glazier

**Affiliations:** Department of Biology, Juniata College, Huntingdon, PA 16652, USA; E-Mail: glazier@juniata.edu; Tel.: +1-814-641-3584; Fax: +1-814-641-3685

**Keywords:** biological regulation, body mass, cellular metabolic rate, mitochondria, scaling, resource-supply limits, systemic effects, tissue composition

## Abstract

The reason why metabolic rate often scales allometrically (disproportionately) with body mass has been debated for decades. A critical question concerns whether metabolic scaling is controlled intrinsically at the intracellular level or systemically at the organismal level. Recently, the relative importance of these effects has been tested by examining the metabolic rates of cultured dermal fibroblast and skeletal muscle cells in relation to donor body mass of a variety of birds and mammals. The lack of a relationship between *in vitro* cellular metabolic rates and body mass suggests that systemic effects, not intrinsic cellular effects are responsible for allometric metabolic scaling observed in whole organisms. Influential resource-transport network theory claims that the most important systemic effect involved is body-size related resource-supply limits to metabolizing cells. However, comparisons of *in vitro* cellular metabolic rates with scaling relationships for *in vivo* (basal) metabolic rates suggest that other systemic effects, such as body-size dependent biological regulation and tissue composition may also have major, perhaps more important effects. Furthermore, systemic effects must ultimately act at the cellular level, for example, by induced variation in the function, structure and intracellular densities of mitochondria. The mechanistic pathways involved require further study.

## 1. Introduction

In many kinds of organisms the rate of respiratory metabolism (*R*) scales with body mass (*M*) according to a simple power function, *R* = *aM^b^*, where *a* is the scaling coefficient (antilog of the intercept in a log-log plot) and *b* is the scaling exponent (slope in a log-log plot). The scaling exponent is often less than 1, thus indicating that larger organisms have lower mass-specific metabolic rates than smaller organisms. Debate about the causes of this negatively allometric (disproportionate) metabolic scaling has occurred for over 80 years, but a consensus has yet to be reached [1,2,3]. One major recurring issue of this debate has been whether the rate of metabolism is set intrinsically at the cellular level or systemically at the whole organism level (or both). Intrinsic effects may include the molecular properties of metabolic pathways and the intracellular structures (e.g., cell membranes and organelles) supporting them [4,5]. Systemic effects may include control by biological (e.g., neuroendocrine) regulatory systems [6,7], body-size related shifts in the proportions of tissues with high *versus* low metabolic rates [2,4,7,8], and (or) resource (oxygen and nutrient) limits to metabolizing cells at the whole organism level [9,10,11].

**Figure 1:**
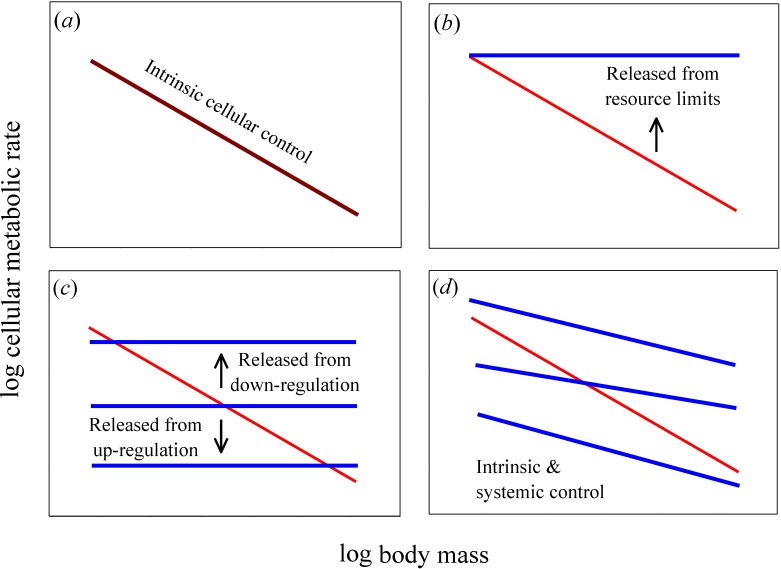
Hypothetical relationships for how log mass-specific cellular metabolic rates *in vitro* (for cultured cells in oxygen- and nutrient-rich media: blue lines) *versus in vivo* (for cells intact in whole organisms: red lines) should scale with log body mass. (***a***) If intrinsic cellular effects predominate, then the scaling of *in vitro* and *in vivo* rates should be the same or nearly so (*i.e.*, the scaling lines should overlap, as shown by a purple line). (***b***) If systemic body-size related resource limits predominate, then *in vitro* rates should exceed *in vivo* rates and the scaling slope for mass-specific *in vitro* rates should be zero or nearly so. (***c***) If systemic effects occur by up or down biological (e.g., neuroendocrine) regulation, then *in vitro* rates should also show no scaling with body mass, but may be greater or lesser than *in vivo* rates. A similar pattern should occur if systemic effects involve decreases in the proportional mass of tissues with high *versus* low metabolic rates as body size increases: *in vitro* rates for each tissue type should show no scaling with body mass, but the rates for different tissue types may be greater or lesser than whole body *in vivo* rates. (***d***) If both intrinsic and systemic factors affect metabolic scaling, the scaling slopes of *in vitro* and *in vivo* rates should both be negative, but differ in magnitude. The elevation of the scaling relationships may also differ between *in vitro* and *in vivo* rates.

A useful test of the relative importance of intrinsic cellular *versus* systemic organismal effects on metabolic scaling relies on a comparison of cellular metabolic rates *in vitro* (in isolated or cultured cells) *versus in vivo* (in the live intact organism) [6,11,12]. If intrinsic effects predominate, cellular metabolic rates and their scaling with body mass should be the same *in vitro* and *in vivo* (Figure 1*a*). By contrast, if systemic effects occur, *in vitro* and *in vivo* cellular metabolic rates and their scaling with body mass should differ significantly (Figure 1*b*–*d*). Moreover, if systemic effects entirely determine the allometric scaling of *in vivo* metabolic rate, then *in vitro* cellular metabolic rates should show no scaling with body mass (*i.e.*, for cell- or mass-specific metabolic rate, *b* = 0, unlike *in vivo* rates where *b* < 0; such as −1/4 or −1/3, as predicted by theory [2,3,11]; Figure 1*b*,*c*). In addition, if systemic resource limits are involved, *in vitro* cellular metabolic rates (for cultured cells in oxygen- and nutrient-rich media) should be greater than (or equal to) *in vivo* rates (Figure 1*b*), whereas if systemic effects involve regulatory control, *in vitro* cellular metabolic rates may be greater or lesser than those *in vivo* (Figure 1*c*). Another possibility is that allometric metabolic scaling results from decreases in the proportional mass of tissues with high *versus* low metabolic rates as body size increases. If this systemic effect predominates, then we can also expect *in vitro* cellular metabolic rates of the same tissue type to show little or no dependence on donor body mass, whereas they should vary significantly among tissue types (as depicted by the horizontal lines with different elevations in Figure 1*c*). If both intrinsic and systemic effects are involved, *in vitro* cellular metabolic rates should show allometric scaling, but not exactly the same as that seen *in vivo* (Figure 1*d*).

Although comparisons between the scaling of *in vitro* and *in vivo* cellular metabolic rates have been made since the 1920s [6,7,12], a resolution to the debate about the relative importance of intrinsic cellular *versus* systemic organismal effects on metabolic scaling has yet to be achieved, principally because of various methodological problems, including inadequate or variable culture techniques and comparisons of heterogeneous tissue types (see [7,12,13]; and also Section 4). Here, I focus on data from recently published studies that minimize these problems by using uniform tissue types and culture conditions. Body-mass scaling of aerobic metabolic rates in cultured or freshly excised liver, skeletal muscle and dermal fibroblast cells are compared with those of intact body cells of birds and mammals (as estimated by mass-specific whole body basal metabolic rates) to test the relative effects of intrinsic cellular *versus* systemic organismal factors on metabolic scaling.

## 2. Materials and Methods

Data for *in vitro* metabolic (oxygen consumption) rates (all at 37 °C) of freshly excised liver cells were taken from [14], of cultured dermal fibroblasts from [15,16,17], and of cultured skeletal muscle cells from [18]. Data for *in vivo* metabolic rates of intact body cells of all tissue types averaged together (*i.e.*, mass-specific basal metabolic rates of birds and mammals with body temperatures averaging near 37 °C) were obtained from the most extensive data bases currently available [19,20]. For comparison, I also examined data on the *in vivo* oxygen consumption rates of liver tissue of five mammal species [21] that were obtained using the arteriovenous oxygen difference technique [22,23]. Metabolic rates were expressed as nmol O_2_ min^−1^ g live mass of cells^−1^. This unit of measurement was derived from literature data expressed in different units using the following assumptions or unit conversions factors (*i.e.*, numbers that allow one unit to be converted into another): dry mass = (protein mass)/0.23 [24]; live (wet) mass = (dry mass)/0.3 [25]; live mass of a dermal fibroblast = 8 ng [17]; ng O_2_ = (nL O_2)_/0.699; nmol O_2_ = (ng O_2_) × 16.67. Scaling relationships were expressed as power functions or as log-log linear equations using least-squares linear regression (LSR). The two most commonly used regression analyses in scaling studies are LSR and reduced major axis (RMA) analyses, but the former is preferred when the Y variable has more error than the X variable, as is usually the case for metabolic rate in relation to body mass [26]. Log-transformation of data was also used because it permits detection of proportional relationships between metabolic rate and mass, a fundamental requirement for allometric analyses [27]. Although the scaling relationship for log mammalian basal metabolic rate *versus* log body mass shows significant curvature [28,29,30], this departure from linearity is slight (increasing *r*^2^ by only 0.003 [31]) and does not affect the analyses in this study.

## 3. Results

**Figure 2:**
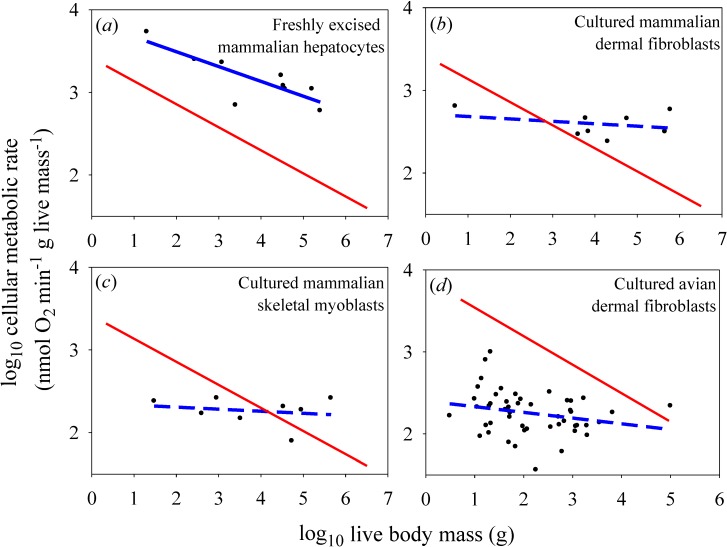
Scaling of log mass-specific cellular metabolic rates *in vitro* (for cultured or freshly excised cells: blue lines) and *in vivo* (for cells intact in whole organisms: red lines) with log body mass. The *in vivo* scaling lines are based on the mass-specific scaling of basal metabolic rate in mammals (***a***, ***b***, ***c***: Y = 2.60(X)^−0.279^; *r*^2^ = 0.959; *p* < 0.0001; N = 639; data from [19]) and birds (*d*: 7.71(X)^−0.348^; *r*^2^ = 0.942; *p* < 0.0001; N = 533; data from [20]). Note that the high *r*^2^ values (for log-log regressions) indicate that these relationships are very strong (only 4.1% and 5.8% of the variation in basal metabolic rate is not explained by body mass in mammals and birds, respectively), and thus very useful as reference lines for comparison. (***a***) Scaling of metabolic rates of freshly excised mammalian liver cells (Y = 3.85 − 0.179(X); *r*^2^ = 0.679; *p* = 0.0063; N = 9; data from [14]). (***b***) Scaling of metabolic rates of cultured mammalian dermal fibroblasts grown in their own serum (Y = 2.71 − 0.029(X); *r*^2^ = 0.093; *p* = 0.46; N = 8; data from [15,16]). (***c***) Scaling of metabolic rates of cultured mammalian skeletal muscle cells (Y = 2.35 − 0.025(X); *r*^2^ = 0.041; *p* = 0.63; N = 8; data from [18]). (***d***) Scaling of metabolic rates of cultured avian dermal fibroblasts (Y = 2.40 − 0.070(X); *r*^2^ = 0.059; *p* = 0.093; N = 49; data from [17]).

**Figure 3:**
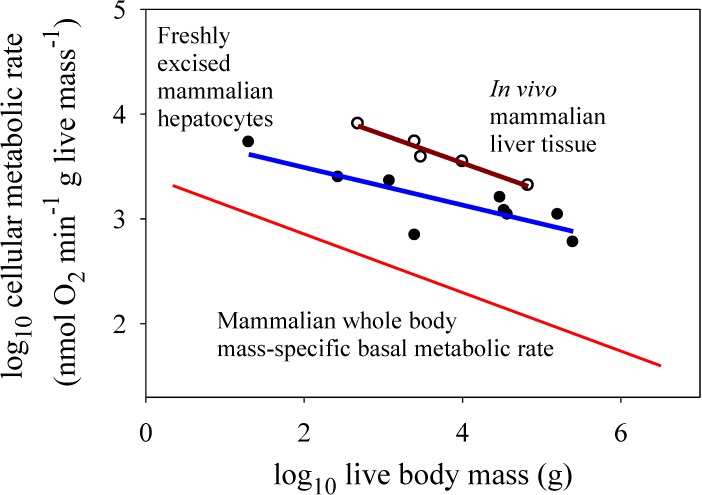
Scaling of log mass-specific metabolic rates of liver cells *in vitro* (for freshly excised cells: solid circles and blue line, Y = 3.85 − 0.179(X); *r*^2^ = 0.679; *p* = 0.0063; N = 9; data from [14]) and *in vivo* (for intact liver tissue: open circles and red line, Y = 4.61 − 0.268(X); *r*^2^ = 0.951; *p* = 0.0047; N = 5; data from [21]) with log body mass. For comparison, the mass-specific scaling of basal metabolic rate in mammals (from Figure 2) is also shown. Note that *in vitro* liver cells have significantly lower metabolic rates than those of *in vivo* liver cells (ANCOVA: *F*_1,11_ = 25.45; *p* = 0.00037). The metabolic scaling slope for *in vitro* liver cells (−0.179 ± 0.110 95% confidence intervals (CI)) is also less steep than that for *in vivo* liver cells (−0.268 ± 0.112), but not significantly so (as seen by the overlapping CI). The scaling slope for *in vivo* liver cells is also not significantly different from that for whole body mass-specific basal metabolic rate (−0.279 ± 0.012).

For the *in vitro* datasets, only the freshly excised liver cells showed a significantly negative relationship between metabolic rate and the body mass of their donor species (Figure 2*a*), but the slope for this relationship (−0.179) was not as steep as that observed for mammalian body cells *in vivo* (−0.279) [the *in vitro* slope lies outside the 95% confidence limits (−0.267 to −0.291) of the *in vivo* slope]. The metabolic scaling slope for freshly excised liver cells was also less steep than that for liver tissue *in vivo* (−0.268), though not significantly so (each slope value was within the 95% confidence limits of the other) (Figure 3). The cultured cells of myoblasts and dermal fibroblasts showed no significant relationships with body mass (scaling slopes not significantly different from zero), in contrast to the highly significant negative relationships exhibited by body cells *in vivo* (−0.279 and −0.348 for mammals and birds, respectively; Figure 2*b–d*).

## 4. Discussion

The scaling relationships for *in vitro* cellular metabolic rates shown in Figure 2 confirm the empirical patterns reported in the papers from which these data were derived [14,15,17,18]. As already argued by several investigators [1,7,11,15,18], the lack of a significant scaling of the metabolic rate of cultured cells with body mass supports the view that metabolic scaling in intact birds and mammals is controlled by systemic organismal factors, rather than by intrinsic cellular factors (compare Figure 2*b–d* with Figure 1*b*,*c*). However, directly (graphically) comparing the scaling of mass-specific cellular metabolic rate *in vitro versus in vivo* (Figure 2 and Figure 3), a technique originally employed by West *et al.* [11], permits further novel insights to be gained, a major purpose of this commentary.

First, the present findings appear to contradict the common belief that the systemic control involved is chiefly body-size-related resource limitation, as predicted by widely cited resource-transport network (RTN) theory (Figure 1*b*; [11]). This conclusion is supported by the observation that *in vitro* metabolic rates of cultured cells are often less than *in vivo* rates of intact cells in the whole organism, contrary to expectation. Many species-specific metabolic rates of cultured myoblasts and fibroblasts are below the regression lines for *in vivo* (basal) metabolic rates (see Figure 2*b–d*). Other observations and calculations also show that cultured cells often show pronounced decreases in metabolic rate compared to cells *in vivo* or those that have been freshly isolated (compare Figure 2*a* with Figure 2*b–d*; also see Figure 3 and [14,16,17]). These findings do not conform to the scaling pattern expected if resource limits in the intact organism were controlling *in vivo* basal metabolic rates (Figure 1*b*). If this were true, *in vivo* basal metabolic rates would always be less than (or equal to) *in vitro* rates, which is clearly not observed, especially for cultured avian dermal fibroblasts (48 of 49 *in vitro* species values are below the regression line for *in vivo* metabolic rates). Furthermore, contrary to RTN theory, metabolic rates of freshly excised liver cells are significantly lower (rather than higher) than those of liver tissue *in vivo* (Figure 3). Although proponents of RTN theory collected literature data on *in vitro* cellular metabolic rates that appear to support the effect of resource limitation on *in vivo* metabolic rates (compare Figure 2 of [11] to Figure 1*b*), these data are problematic because they include metabolic rates from several kinds of cells that were cultured under diverse conditions. Furthermore, many of the cell types were cancerous [32], and thus likely had metabolic properties different from that of normal cells [33]. The high phenotypic plasticity of the structure and function of the mammalian circulatory system in response to changes in metabolic demand also suggests that RTNs are unlikely to constrain *in vivo* basal metabolic rates [6,7].

Second, the present results appear to be more consistent with systemic control by body-size related biological regulation and (or) tissue composition (as depicted in Figure 1*c*). Metabolic rates of cultured cells are either less or more than *in vivo* rates (Figure 2*b–d*), as expected if the latter were affected by either up- or down regulation, respectively (Figure 1*c*). Greater proportional mass of tissues with low *versus* high metabolic rates in larger species of birds and mammals (see [7,8]) may also be involved, but this hypothesis cannot explain why *in vivo* or freshly isolated cells of the same tissue type often show negative allometry of metabolic rate (e.g., liver cells: Figure 2*a* and Figure 3; many other examples for other tissue types are reviewed in [1,2,6,7,12,21]). However, the metabolic scaling slopes of freshly isolated cells are usually less steep than those for *in vivo* metabolic rates (e.g., Figure 2*a* and Figure 3), thus inspiring some investigators to implicate the effects of both intrinsic cellular factors and body-size dependent tissue composition on metabolic scaling [4,8]. In any case, much of the differences in metabolic rates observed among different tissue types may be ultimately due to biological regulation (see review in [7]). Therefore, I suggest that biological (e.g., neuroendocrine) regulation is a major systemic factor causing body-mass scaling of basal metabolic rate in birds and mammals [34]. Biological regulation may not only directly affect metabolic rate, but also indirectly by its effects on or responses to body-size dependent tissue composition and resource limitation, a hypothesis requiring further testing.

Third, another possible interpretation of the lack of body-mass scaling of metabolic rate in isolated cultured cells is that outside of the body they no longer need to be engaged in various routine physiological functions, thus causing them to metabolize at a uniformly minimal level required for survival [18]. However, this hypothesis of “cultured quiescence” does not exclude systemic regulatory effects that may be responsible for differences in activity between cells *in vivo versus* *in vitro*. Furthermore, this hypothesis is inconsistent with the observation that the metabolic rates of cultured cells may be either above or below that of *in vivo* cells (Figure 2*b–d*).

Fourth, one may hypothesize that the type and quantity of substrate offered to cultured cells has affected their metabolic rates and thus their magnitude relative to *in vivo* metabolic rates. Since all of the *in vitro* metabolic data used in my analyses come from studies that used uniform culture conditions, this hypothesis by itself would predict that *in vitro* metabolic rates should show uniform differences from *in vivo* rates, or nearly so, regardless of donor body size [35]. However, this pattern is clearly not observed: differences between *in vitro* and *in vivo* metabolic rates depend strongly on body size (Figure 2*b–d*), and thus are more likely the result of body-size related variation in the regulation of *in vivo* metabolic rates. It also seems unlikely that other kinds of possible effects resulting from excising and culturing cells *in vitro* could explain the strongly body-size related differences observed between *in vitro* and *in vivo* cellular metabolic rates.

Fifth, the type of biological regulation involved in the metabolic scaling of birds and mammals is most likely body temperature regulation, at least in part. Maintenance of a constant high body temperature in endothermic animals requires that heat loss be exactly balanced by heat production. Since heat loss depends on surface area, which scales with body mass to the 2/3 power in mammals [36], both heat loss and compensating metabolic heat production should also scale to the 2/3 power. This simple argument was first proposed approximately 175 years ago, but unfortunately it has often been regarded as being discredited by investigators seeking a single universal mechanism for metabolic scaling [7]. Although it may not apply to ectothermic organisms with variable body temperatures, this does not mean that it is irrelevant for homeothermic endotherms. The hypothesis that thermoregulation plays an important role in endothermic metabolic scaling is supported by several lines of evidence (reviewed in [7]), including that (1) the scaling slopes for heat loss in mammals [37] and for basal and cold-induced metabolic rates in birds and mammals (especially small mammals) all approximate 2/3 [38], as predicted; (2) huddled small mammals down-regulate their metabolism in such a way that metabolic scaling continues to match the exposed surface area (and thus presumably heat loss) of the entire huddle [7], and (3) many birds and mammals can actively up- and down-regulate their metabolism during periods of torpor or hibernation. Remarkably, during deep hibernation, down-regulation of metabolism results in a marked shift of interspecific metabolic scaling in mammals from being allometric, when body temperature is controlled at a high constant level, to isometric (*i.e.*, directly proportional with body mass), when homeothermy is no longer maintained [7,39]. In effect, the metabolic scaling of hibernating mammals becomes similar to that of ectothermic animals with variable body temperatures and relatively low metabolic rates that are not matched to surface-area related heat loss, but rather more nearly to volume related tissue maintenance [39]. Collectively these observations and those presented in Figure 2 provide strong evidence that biological regulation plays an important role in the metabolic scaling of birds and mammals, albeit in the context of body-size dependent structural constraints—*i.e.*, surface area to volume relationships that affect body heat loss (also see [7,30,39] and for a contrasting view [40,41]).

Sixth, biological regulation likely influences the metabolic scaling of birds and mammals (and probably other eukaryotic organisms) via effects on the function, structure and (or) intracellular densities of mitochondria. The allometric scaling of metabolic rate in freshly isolated mammalian hepatocytes (Figure 2*a*) is matched by parallel or nearly parallel scaling of the densities, surface areas and proton-leak rates of mitochondria [14]. However, like the allometric scaling of metabolic rate, the allometric scaling of mitochondrial densities, proton-leak rates and enzyme activities is lost in cultured cells that are released from systemic influences ([15,17,18]; also compare to predictions of [11]).

Seventh, the present observations do not rule out cellular effects on metabolic rate and its scaling with body mass. As noted above, the mechanism of action of metabolic regulation must occur, at least in part, at the intracellular (biochemical) level. The observation of allometric scaling in freshly isolated cells that, however, differs both in slope and elevation from that of *in vivo* body cells suggests that both intrinsic and systemic factors are involved in metabolic scaling (compare Figure 2a and Figure 3 with Figure 1*d*; also see [6,7]). However, the “intrinsic” differences seen in freshly isolated cells [14] may be the result of lingering systemic effects that gradually disappear during long-term culture, as observed (Figure 2*b–d*: also see [15,17,18]). Perhaps in the absence of direct systemic (e.g., hormonal) influences, the number and metabolic activity level of mitochondria within isolated cells gradually shifts to suit a cell’s changed functional demands and survival needs in a laboratory culture environment. Intrinsic effects may also be involved in causing residual variation in cellular metabolic rates unexplained by body-size differences, which can be quite large, as observed in avian dermal fibroblasts (Figure 2*d*). However, even these differences may relate to systemic properties of organisms (e.g., differences in life-history traits, such as life span or growth rate). In fact, among bird species the metabolic rate of dermal fibroblasts is significantly correlated with whole-body growth rate [17].

Another possibility is that body-size related differences in cell size affect the metabolic scaling observed in birds and mammals. According to the cell-size model [42,43], negatively allometric metabolic scaling may arise from positive scaling of cell size with body size (scaling slope > 0). Larger cells have smaller surface area to volume ratios, thus potentially reducing the mass-specific supply of resources fueling cellular metabolic processes and the release of wastes produced by them. The mass-specific metabolic demand for surface-area related ionic regulation is also expected to be smaller in larger cells [44]. As a result, large animals with relatively large cells should have lower metabolic rates than small animals with relatively small cells. However, cell size is very similar in mammals ranging in size from tiny mice to huge elephants (cell size scales with mammalian body mass to only the 0.03 to 0.05 power: [45,46,47]). Therefore, the cell-size model was not considered here as a possible explanation of interspecific allometric metabolic scaling in birds and mammals, though it may be relevant for ontogenetic metabolic scaling [2,48,49,50,51] and interspecific metabolic scaling observed in other kinds of organisms [7,49].

A possible criticism of my analyses is that metabolic data for hepatocytes, myoblasts and dermal fibroblasts may not reflect that of other types of body cells. For example, liver cells tend to show higher mass-specific metabolic rates *in vivo* than the body as a whole (Figure 3). However, liver metabolic rates are highly sensitive to an animal’s physiological condition. Since the liver is importantly involved in food processing, its metabolic rate is strongly related to the food intake and nutritional state of an animal [22,52,53,54,55,56]. In cattle, liver oxygen uptake can vary up to an order of magnitude depending on level of food intake and processing [22]. Increases in liver metabolism have also been shown to be a major contributor to diet-induced thermogenesis (specific dynamic action) in rats [52]. During fasting liver cells show lower metabolic rates that are closer to basal levels for the body as a whole [22,55,56]. In any case, how the metabolic rates of various kinds of cells in various tissues relate to each other and to whole body metabolic rate requires further study (for some insight, see [8,21]). Many kinds of freshly isolated tissues (including not only liver and muscle, but also brain, kidney, spleen and lung) show similar allometric scaling with donor body mass (see discussion and references cited in [12,14,15]). In addition, although very different physiologically, cultured myoblasts and fibroblasts show similar patterns of metabolic scaling (Figure 2*b–d*; also see [17]). Myoblasts and fibroblasts are especially useful for *in vitro* scaling analyses because they readily replicate to produce homogeneous cell cultures [15,17,18]. Nevertheless, it would be useful to explore how the metabolic rates of other kinds of cultured cells scale with body mass.

## 5. Conclusions

My analyses are consistent with the view that biological regulation is importantly involved in metabolic scaling, which although proposed long ago [6,7], has been rarely tested. Metabolic rate and its scaling with body mass appear not to be solely functions of energetic or physical constraints, as proposed by resource-centered theory (e.g., [11,57]), but rather are also influenced by information-based regulatory systems. Further research is needed to pinpoint the regulatory mechanisms that are actually involved [7]. A complete understanding of why the pace of living processes (including metabolic rate) varies requires an appreciation of the importance of both resource- and information-based control, two major features of all life [7,58].

I hope that my tentative, somewhat provocative conclusions will stimulate others to further test the relative roles of body-size related biological regulation, tissue composition, resource-supply limits and various structural constraints (e.g., body surface area to volume ratios, physical properties of resource transport systems, and the relative size and number of body cells) on metabolic scaling, not only in birds and mammals, but also in many other kinds of organisms.
